# Umbilical *lichen planus* induced by nivolumab^☆^

**DOI:** 10.1016/j.abd.2021.09.022

**Published:** 2023-05-08

**Authors:** Luisa Martos-Cabrera, Iñigo Lladó, Paloma Fernández-Rico, Beatriz Butrón-Bris, Pedro Rodríguez-Jiménez

**Affiliations:** a Department of Dermatology, Hospital Universitario de la Princesa, Madrid, Spain; b Department of Pathology, Hospital Universitario de la Princesa, Madrid, Spain

Dear Editor,

Skin adverse events are the most common side effects under anti-PD1 immunotherapy. They usually develop early in the course of treatment and do not require interruption.^1^ However, alternative clinical presentations may be observed.^1^, ^2^ The authors encourage you to report them as they may improve knowledge of both, drugs and disease. Hence, the authors present an unusual case of lichen planus (LP) in the umbilicus after the fifteenth dose of nivolumab.

A 77-year-old Caucasian female presented with a 3-month history of a red, scaly, itchy, asymmetrical patch located at the umbilicus (Fig. 1). The patch had developed over a few days, persisted since, and no other lesions were found upon complete mucocutaneous examination. Under dermoscopy, white streaks on a violaceus background could be observed (Fig. 2). The patient had a past personal history of metastatic melanoma under treatment with nivolumab 240 mg every two weeks until the date. She started it 20 months prior to the appearance of the umbilical patch. A 4 mm punch biopsy was performed.

**Fig. 1:**
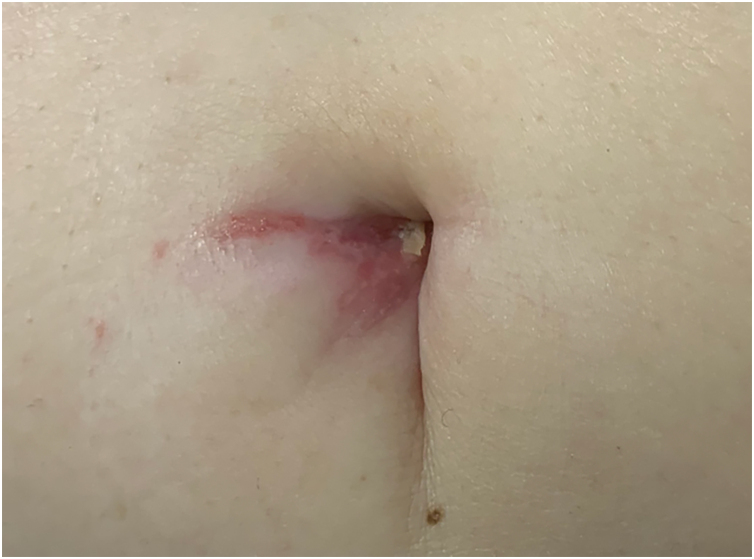
A single violaceous patch with a fine reticulate pattern of dots and lines (Wickham’s striae) on the umbilicus

**Fig. 2:**
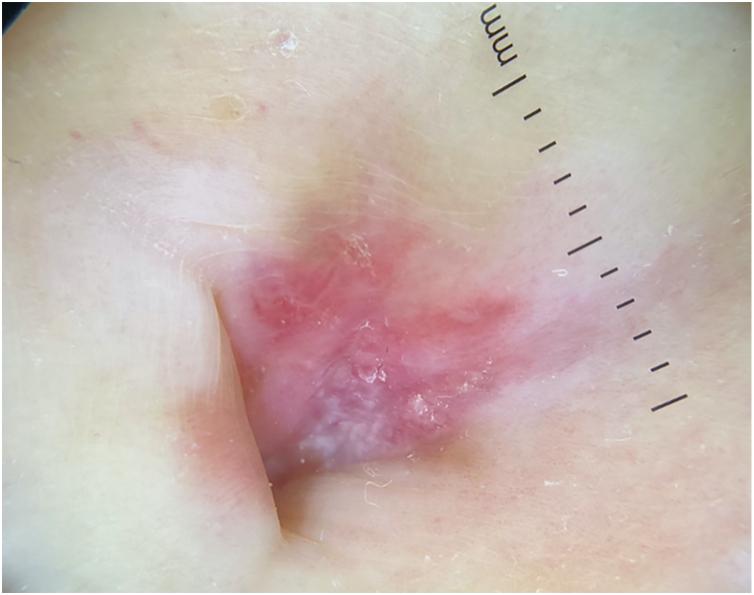
Characteristic white streaks on a violaceus background could be observed under dermoscopy

Histology showed hyperkeratosis and cytoid bodies with a bandlike inflammatory cell infiltrate composed of lymphocytes, histiocytes, and occasional eosinophils in the papillary dermis (Fig. 3). The features favored a diagnosis of LP.^3^ Serologies for VHB and VHC were negative. She has been prescribed clobetasol propionate 0.05% ointment for 4 weeks with partial response.

**Fig. 3:**
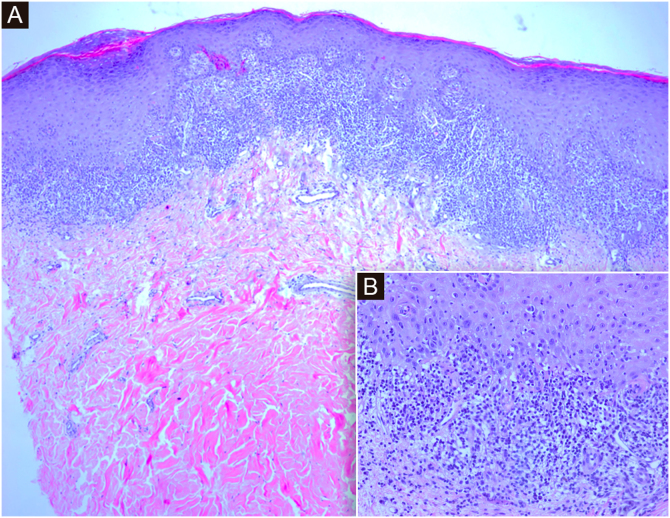
(A) Histological sections show an epidermis with orthokeratotic hyperkeratosis with focal parakeratosis, acanthosis with hypergranulosis, and a dense subepidermal band inflammatory infiltrate with interface dermatitis. (Hematoxylin & eosin, ×4). (B) At higher magnification, lymphocytic interface dermatitis with vacuolar degeneration of the basal layer and necrotic keratinocytes in the epidermis. (Hematoxylin & eosin, ×20)

PD1 pathway inhibits T-cell activation keeping normal immune response balanced.^1^, ^2^ Several malignant cells activate PD1 favoring immune escape. Anti-PD1 therapy seeks to activate the immune system in order to kill malignant cells.^1^, ^2^ On the other hand, T-cells play an important role in the pathogenesis of LP.^3^ Under this scope, a T-cell activation induced by immunotherapeutic agents blocking PD1 could possibly contribute, along with other stimulating factors, to LP development.^1^, ^2^, ^3^ Lichenoid skin reactions are well-known side effects of anti-PD1 therapy.^1^, ^2^, ^4^ The incidence of related lichenoid eruption is probably underestimated due to its sporadic publication.^1^, ^2^ Clinically, it is normally presented as multiple, discrete, erythematous, violaceous papules and plaques,^3^ thus the patient's umbilical exclusive location seems a rarity. In fact, the authors have only found one case of LP affecting this area, but not exclusively, in one patient suffering from vitiligo as well.^5^ Interestingly, relatively uncommon forms of other skin conditions exacerbated by anti-PD1 therapy have been described.^1^, ^2^, ^4^ Although normally presented in the first months after treatment, some authors suggested that the onset of lichenoid eruptions may be delayed compared with other skin reactions.^4^ Indeed, Wang et al. reported that cutaneous adverse reactions may present with delayed onsets and even after discontinuation of therapy.^4^ However, the authors are aware that another unknown stimulating factor different from nivolumab could not be completely ruled out.

In conclusion, the authors present a unique case of LP due to the exclusive umbilical location in a patient treated with nivolumab to highlight the potential delayed and alternative clinical presentation of anti-PD1 reactions and that specialized dermatologist consultation should be considered mandatory for accurate diagnosis and best treatment.

## Financial support

None declared.

## Authors’ contributions

Luisa Martos-Cabrera: Have made substantial contributions to conception and design, or acquisition of data, or analysis and interpretation of data; and have been involved in drafting the manuscript or revising it critically for important intellectual content.

Iñigo Lladó: Have made substantial contributions to conception and design, or acquisition of data, or analysis and interpretation of data; and have been involved in drafting the manuscript or revising it critically for important intellectual content; have made substantial contributions to acquisition of data and have been involved in given final approval of the version to be published.

Paloma Fernández-Rico: Have made substantial contributions to conception and design, acquisition of data and analysis and interpretation of data.

Beatriz Butrón-Bris: Have made substantial contributions to conception and design, acquisition of data and analysis and interpretation of data.

Pedro Rodríguez-Jiménez: Have made substantial contributions to conception and design, or acquisition of data, or analysis and interpretation of data; and have been involved in drafting the manuscript or revising it critically for important intellectual content; given final approval of the version to be published.

## Conflicts of interest

None declared.
